# Stevens-Johnson Syndrome Due to Influenza Vaccination

**DOI:** 10.7759/cureus.9405

**Published:** 2020-07-26

**Authors:** Jonathan Tong, June Chan

**Affiliations:** 1 Literature, Science, and the Arts, University of Michigan, Ann Arbor, USA; 2 Radiation Oncology, Ascension Providence Hospital, Novi, USA

**Keywords:** stevens johnson syndrome, toxic epidermal necrolysis, drug-related side effects and adverse reactions

## Abstract

Influenza is a common virus that affects millions of people every year. The influenza vaccine decreases morbidity and mortality associated with influenza and is generally well tolerated. Stevens-Johnson syndrome (SJS) is a rare disorder of the skin and mucous membranes. We report the second known case of SJS occurring after an influenza vaccination alone without any other associated drug exposure. This corroborates the possibility of the influenza vaccine alone causing SJS. Despite the initial adverse reaction, the patient made a full recovery. Although the disease can be associated with vaccinations, the benefits of receiving the vaccinations outweigh the potential harms.

## Introduction

Stevens-Johnson syndrome (SJS) is a rare disorder of the skin and mucous membranes that occurs in about one to three cases every million persons each year [1]. This serious disorder is characterized by flu-like symptoms, red or irritated skin, and blisters. Although cases related to vaccinations have been reported, there has been only one case reported due to the influenza vaccine only in a 24-year-old female [1]. There has been a second case reporting SJS occurring after receiving the influenza vaccine, but this was given in combination with fluvoxacillin, so it does not corroborate the first case [2]. We report the second known case of SJS occurring after an influenza vaccination alone, which corroborates the possibility of the influenza vaccination alone causing SJS.

## Case presentation

**Figure 1:**
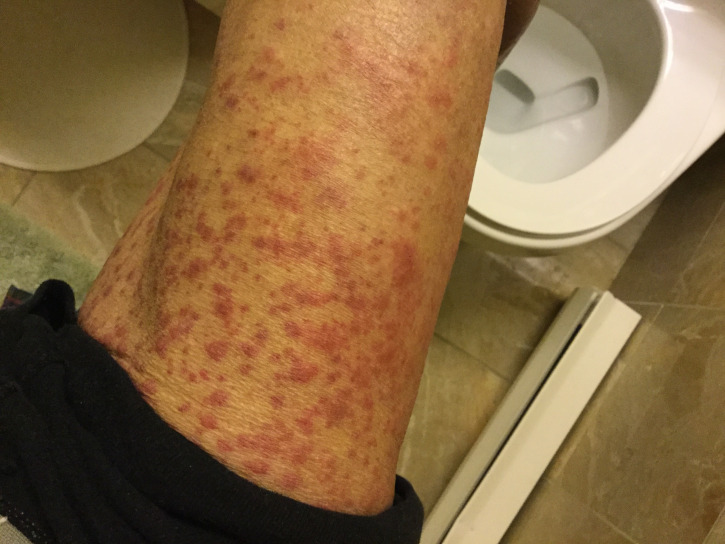
Thigh four days after influenza vaccination Multiple purpuric macules are present. There is a lack of serious skin detachment or blistering.

**Figure 2:**
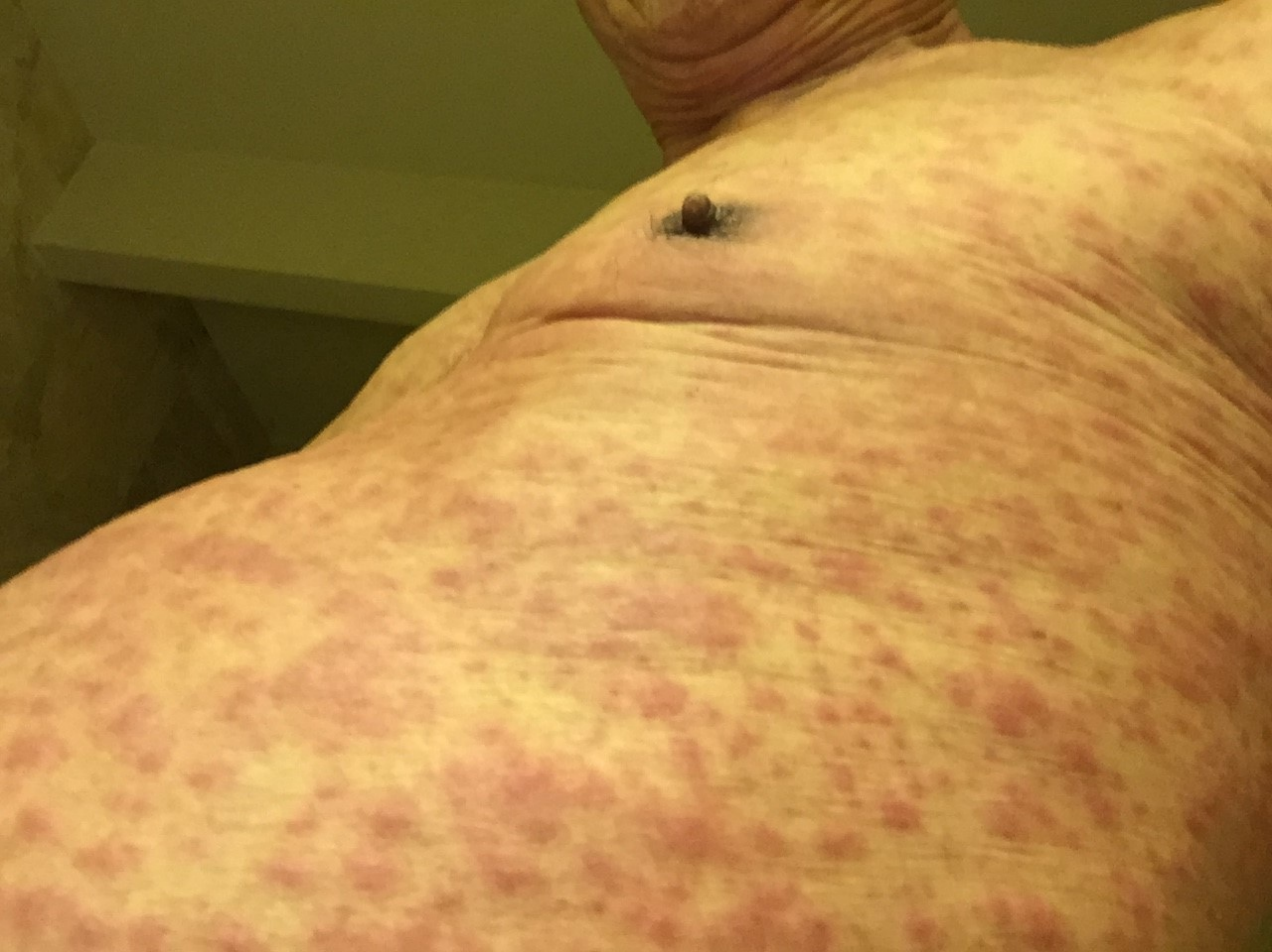
Trunk four days after influenza vaccination Diffuse presentation of coalescing macules across the trunk. Lack of serious skin detachment.

**Figure 3:**
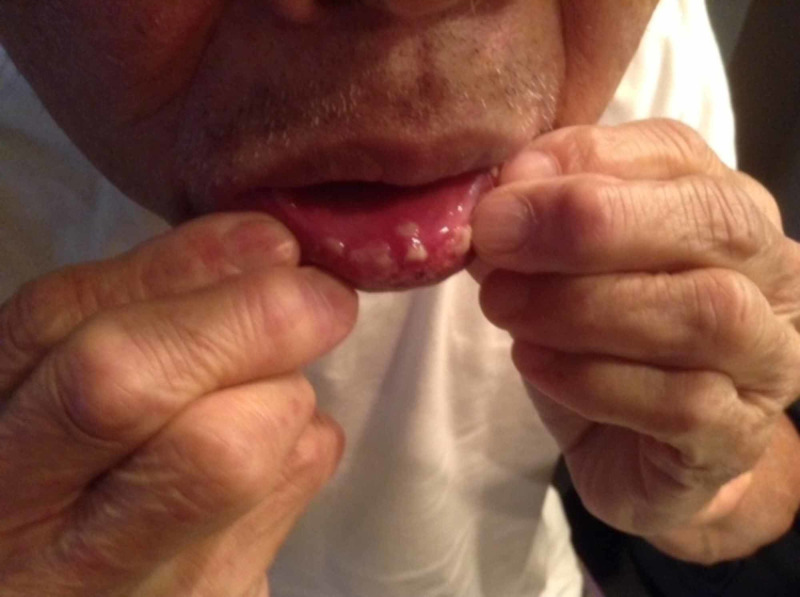
Lower lip eight days after vaccination Multiple coalescing papules present on oral mucosal membrane.

The patient was an 82-year-old male with a past medical history of successfully treated prostate cancer and hypertension. His medications included atorvastatin, candesartan, metoprolol, and aspirin taken with no dose changes for at least two years. He received the influenza vaccine Influvac® (Abbott), which he had done annually without any complications. The patient had no symptoms on the day of the injection. One day after the vaccination, he noted mild pruritus in the evening. A rash was noted in the chest and back, and he was started diphenhydramine 25 mg orally as well as diphenhydramine cream. Four days after the vaccination, the rash had spread to his arms and legs (Figures 1, 2). Five days after the vaccination, the rash spread to the face, and he went to a walk-in clinic. Upon examination, his dose of diphenhydramine was increased to 50 mg. That afternoon, the patient’s wife noticed facial swelling and brought him into the emergency room of a major city hospital. The doctor noted an inner lip ulcer and diagnosed it as mild Stevens-Johnson syndrome based on the morbilliform rash on his body that had spread to the patient’s face. He was admitted to the hospital for two nights. A dermatologist verified the diagnosis of SJS and prescribed cetirizine (10mg daily for seven days). The patient had an estimated 85% body surface area affected with a SCORe of Toxic Epidermal Necrosis(SCORTEN) score of 1 [1]. Oral steroids were suggested, but the patient declined to take them. One day after discharge (eight days after the influenza vaccination), he noted the appearance of more ulcers on his bottom lip (Figure 3). Nine days after the influenza vaccination, he saw another dermatologist who recommended an oral mouthwash for symptomatic relief. Gradually, the skin rash improved, but the oral ulcers worsened until about nine days after vaccination and improved by 13 days after vaccination. The patient felt he was mostly recovered about a month after the vaccine. Other than the flu vaccine, patient had not taken any new medications, new supplements, or new foods prior to the development of the itching and skin rash.

## Discussion

Even though SJS is rare with an annual incidence of one to three reported cases per million people [1], there have been many reported cases of SJS since its discovery in 1922 [3]. Over 200 different medications have been identified as causes of SJS, most commonly sulfonamide antibiotics, aromatic anticonvulsants, beta-lactam antibiotics, nevirapine, abacavir, non-steroidal anti-inflammatory medications, allopurinol, lamotrigine, tetracyclines, quinolones, and others [4]. These drugs impact various physiological factors that can lead to the development of SJS. Cytotoxic T cells and natural killer cells have been associated with SJS due to the release of granulysin which destroys cells in the skin and mucous membrane. The cell death that occurs in these areas is generally identified as apoptosis as a result of dysregulation in specific transmembrane protein pathways. The most extensively investigate pathway is that of Fas. This pathway involves the binding of a soluble ligand that induces a caspase cascade leading to the destruction of the cell. Typically SJS occurs in a younger population and is a response to multiple drugs [4].

Mycoplasma pneumonia has been documented in a few cases [5]. SJS has also been associated with Herpes simplex virus [6]. Fleming in 2011 reported a case where a 65-year-old female with a breast abscess was given oral flucloxacillin, then on day 10 of the treatment, she was given the inactivated influenza vaccine Fluvirin® (Novartis) [2]. About 24 hours later, she developed SJS. Because of the abscess infection as well as dual exposures to the antibiotic and the vaccine, the definitive cause could not be determined.

There are several main differential diagnoses for SJS. Acute generalized exanthematous pustulosis (AGEP) is characterized by pinhead-sized pustules that were not present in this case. AGEP is more commonly seen in middle-aged adults and only involves mucosal membranes in roughly 20%-25% of cases [7]. Erythema multiforme is another disease that presents similarly to SJS with skin lesions. These lesions, however, are most commonly associated with the herpes simplex virus and usually do not involve mucosal membranes [4]. In addition to this, the lesions are typically localized or raised atypical targets [8]. Toxic epidermal necrolysis (TEN) presents similarly to SJS, usually beginning with fever-like symptoms followed by cutaneous and mucous membrane lesions. However, the definition of each differs in the extent of skin detachment. SJS encompasses <10% of skin involvement while TEN encompasses >30% of the skin [8]. This patient has <10% skin detachment which is consistent with SJS.

SJS is very rarely associated with vaccination exposure only. A study of the Vaccine Adverse Event Reporting System noted over a nine-year period, there were 96,951 distinct adverse event reports noted of which six cases were either SJS or TEN following exposure to vaccination only [1]. Chopra reported SJS in a 19-year-old male military reservist who received immunizations to smallpox, anthrax, and tetanus then developed SJS twenty days after the vaccinations [9]. SJS was reported following two cases of measles vaccination [10]. To our knowledge, SJS has been previously reported in one case following exposure to only the inactivated seasonal influenza vaccine. Our case thus corroborates the possibility of SJS occurring secondary to the influenza vaccine only.

**Table 1:** SJS/TEN cases reported after vaccination administration DTP - diphtheria, tetanus, whole-cell pertussis; Hib - Haemophilus influenzae type b bacterial; MMR - measles, mumps, rubella; OPV - oral polio vaccine, PAP - papanicolaou smear Sources: [1, 2, 9, 10]

Age (years)	Gender	Vaccines and medicine	Onset	Illnesses and drugs	Severity
0.5	F	Hepatitis B	1 day	Positive eyelid culture for Branhamella catarrhalis, Staphylococcus aureus, Streptococcus pneumoniae. Acetaminophen was given after the onset of rash.	Hospitalized
.8	M	Measles	1 day	Steroids and antibiotic therapy were given one day and two days after vaccination, respectively.	Hospitalized
1.3	M	Hib, MMR	22 days	History of torticollis and exposure to strep throat. Penicillin administered after the onset of rash.	Multiple clinic visits
2.3	M	Varicella	1 day	Diphenhydramine one day after vaccination. Previously developed erythema multiforme after amoxicillin/clavulanate after trimethoprim-sulfamethoxazole.	Hospitalized
2.3	M	DTP, Hib, MMR, OPV	7 days	Diphenhydramine and amoxicillin were given seen days after vaccination.	Hospitalized
3.7	M	Hepatitis B	1 day	No previous medication or illness. Acetaminophen was given after onset of rash.	Hospitalized
19	M	Varicella, anthrax, and tetanus	20 days	Allergy to nickel with no medications noted.	Hospitalized
24.1	F	Influenza	Same day	History of acne, abnormal PAP smear, and medroxyprogesterone injection for birth control.	Hospitalized
65	F	Influenza and flucloxacillin	1 day	History of diabetes. Flucloxacillin was given 10 days prior to vaccination for breast abscess.	Hospitalized

Influenza is a common virus that affects millions of people every year. From 1976 to 2007, estimated flu-related deaths in the United States since 2010 have ranged from 12,000 to 56,000 [11]. The vaccine decreases morbidity and mortality associated with influenza. During the 2011-2012 flu season, influenza vaccination was associated with a 71% reduction in flu-related hospitalizations among adults of all ages and a 77% reduction among adults 50 years of age and older [12]. Since this case illustrates a very rare complication of the influenza vaccine, the benefits of the influenza vaccine clearly far outweigh its potential harm. Therefore this case should not deter people from receiving the influenza vaccination.

## Conclusions

SJS is a rare condition typically associated with oral drugs and less commonly associated with infections. It is rarely associated with vaccinations and has been reported after the influenza vaccine alone in one case. This is the second reported case where SJS has been associated with an influenza vaccination alone which supports the possibility that it can result solely from the influenza vaccine. Although the disease can be associated with vaccinations, the benefits of receiving the vaccinations outweigh the potential harms.

## References

[1] Ball R, Ball LK, Wise RP, Braun MM, Beeler JA, Salive ME (2001). Stevens-Johnson syndrome and toxic epidermal necrolysis after vaccination: reports to the vaccine adverse event reporting system. Pediatr Infect Dis J.

[2] Fleming JD, Fogo AJ, Creamer DJ (2011). Stevens-Johnson syndrome triggered by seasonal influenza vaccination and flucloxacillin: a pathogenetic hypothesis. Eur J Dermatol.

[3] Stevens AM, Johnson FC (1922). A new eruptive fever associated with stomatitis and ophtalmia; report of two cases in children. Am J Dis Child.

[4] Kohanim S, Palioura S, Saeed HN (2016). Stevens-Johnson syndrome/toxic epidermal necrolysis - a comprehensive review and guide to therapy. I. Systemic Disease. Ocul Surf.

[5] Olson D, Watkins LK, Demirjian A (2015). Outbreak of mycoplasma pneumoniae-associated Stevens-Johnson syndrome. Pediatrics.

[6] Forman R, Koren G, Shear NH (2002). Erythema multiforme, Stevens-Johnson syndrome and toxic epidermal necrolysis in children: a review of 10 years' experience. Drug Saf.

[7] Feldmeyer L, Heidemeyer K, Yawalkar N (2016). Acute generalized exanthematous pustulosis: pathogenesis, genetic background, clinical variants and therapy. Int J Mol Sci.

[8] Roujeau JC (1994). The spectrum of Stevens-Johnson syndrome and toxic epidermal necrolysis: a clinical classification. J Invest Dermatol.

[9] Chopra A, Drage LA, Hanson EM, Touchet NL (2004). Stevens-Johnson syndrome after immunization with smallpox, anthrax, and tetanus vaccines. Mayo Clin Proc.

[10] Hazir T, Saleem M, Abbas KA (1997). Stevens-Johnson syndrome following measles vaccination. J Pak Med Assoc.

[11] CDC CDC (2018). Key facts about seasonal flu vaccine. Available.

[12] Talbot HK, Zhu Y, Chen Q, Williams JV, Thompson MG, Griffin MR (2013). Effectiveness of influenza vaccine for preventing laboratory-confirmed influenza hospitalizations in adults, 2011-2012 influenza season. Clin Infect Dis.

